# Altered amygdala effective connectivity in migraine without aura: evidence from resting‐state fMRI with Granger causality analysis

**DOI:** 10.1186/s10194-021-01240-8

**Published:** 2021-04-15

**Authors:** Xiaobin Huang, Di Zhang, Peng Wang, Cunnan Mao, Zhengfei Miao, Chunmei Liu, Chenjie Xu, Xindao Yin, Xinying Wu

**Affiliations:** 1 Department of Radiology, Nanjing First Hospital, Nanjing Medical University, No.68, Changle Road Nanjing, 210006 Nanjing, Jiangsu Province China; 2 Department of Neurology, Nanjing First Hospital, Nanjing Medical University, No.68, Changle Road, 210006 Nanjing, Jiangsu Province China; 3 Department of Pain Treatment, Nanjing First Hospital, Nanjing Medical University, No.68, Changle Road, 210006 Nanjing, Jiangsu Province China

**Keywords:** Migraine, Granger causality analysis, Amygdala, Pain modulation

## Abstract

**Background:**

Granger causality analysis (GCA) has been used to investigate the pathophysiology of migraine. Amygdala plays a key role in pain modulation of migraine attack. However, the detailed neuromechanism remained to be elucidated. We applied GCA to explore the amygdala-based directional effective connectivity in migraine without aura (MwoA) and to determine the relation with clinical characteristics.

**Methods:**

Forty-five MwoA patients and forty age-, sex-, and years of education-matched healthy controls(HCs) underwent resting-state functional magnetic resonance imaging (fMRI). Bilateral amygdala were used as seed regions in GCA to investigate directional effective connectivity and relation with migraine duration or attack frequency.

**Results:**

MwoA patients showed significantly decreased effective connectivity from right amygdala to right superior temporal gyrus, left superior temporal gyrus and right precentral gyrus compared with HCs. Furthermore, MwoA patients demonstrated significantly decreased effective connectivity from the left amygdala to the ipsilateral superior temporal gyrus. Also, MwoA patients showed enhanced effective connectivity from left inferior frontal gyrus to left amygdala. Effective connectivity outflow from right amygdala to right precentral gyrus was negatively correlated to disease duration.

**Conclusions:**

Altered directional effective connectivity of amygdala demonstrated that neurolimbic pain networks contribute to multisensory integration abnormalities and deficits in pain modulation of MwoA patients.

## Introduction

Migraine is a disabling primary headache disorder and was ranked third–highest cause of disability worldwide in both males and females under the age of 50 years[1]. Migraine without aura(MwoA) is the most common type of migraine. It is a recurrent headache disorder menifesting in attacks lasting 4–72 h with typically unilateral in location, pulsating in quality, moderate to severe in intensity, aggravated by physical activity, and associated with nausea and light and sound sensitivity (photophobia and phonophobia)[2, 3]. However, the neuromechanism of the MwoA, which would help to choose optimal treatment to improve the life quality of patients, is remaining elucidated.

Amygdala, which is a large grey matter complex in neurolimbic system, plays a key role in pain modulation during a migraine attack[4–7]. Aberrant connectivities between amygdala and somatosensory cortex regions in migraineurs were observed[8]. Seed-based whole-brain correlation method showed migraine patients have disrupted limbic system (amygdala and hippocampus) functional connectivity to pain-related cortex regions of modulatory and encoding[9]. However, most of previous studies about the effective connectivity in neurolimbic system omit the directional influence of the cerebral functional cortex on amygdala. The directional influence of amygdala might reflect pain modulation principle in MwoA. Fortunately, Granger causality analysis (GCA) is an advanced fMRI data processing method to investigate the top-down control between the cerebral functional cortex and the amygdala [10–12]. The specific intrinsic brain effective connectivity among pain-related networks in MwoA patients are also affected after long-term migraine attacks[13, 14]. Though many studies have proved that migraine is associated with changes in functional connectivity between different regions, no studies discern the directionality or specificity of the disrupted connections related to amygdala.

To address this issue, we applied GCA to identify differences in the direction of functional connectivity between MwoA patients and healthy controls. We selected the bilateral amygdala as seed regions to elucidate mechanism of neurolimbic pain-modulation in migraine pathogenesis. We hypothesized that GCA and functional connectivity of these regions would be disrupted in migraine patients. Moreover, these alterations would be associated with clinical characteristics such as disease duration or attack frequency. To our knowledge, this is the first study to use GCA to unravel the effective connectivity within the limbic system in migraine patients.

## Method

### Participants

According to the International Classification of Headache Disorders, Third Edition (beta version) (ICHD-3beta), 45 MwoA patients were recruited in this study between May 2018 and June 2020. All subjects were right-handed. None of the patients took any preventive medications. To avoid possible interference of pain or pharmacological substances on BOLD signal fluctuation, patients were migraine free for at least 3 days before the fMRI scan. The exclusion criteria included psychotic disorder, major physical illness, e.g. cancer, psychoactive, preventive or chronic medication and contraindications to MRI. Meanwhile, 40 age-, sex-, and years of education-matched healthy subjects were recruited as healthy controls (HCs). The inclusion criteria were having no personal or family history of migraine or any other type of headaches, and free from any chronic medical condition. To minimize hormonal influences on cortical excitability, all female participants were included at mid-cycle and excluded if pregnant or breast-feeding. All participants completed the Hamilton Anxiety Scale (HAMA), Hamilton Depression Scale (HAMD), Montreal Cognitive Assessment (MoCA), Headache Impact Test-6 (HIT-6), and Migraine Disability Assessment Questionnaire. This study was approved by the Institutional Review Board of our university. All participants provided written informed consent before undergoing MR scan.

### Imaging methods

A 3.0 Tesla magnetic resonance imaging scanner (Ingenia, Philips Medical Systems, Netherlands) with an 8-channel head coil was used in this study. Functional images were obtained axially using a gradient echo-planar imaging sequence as follows: repetition time (TR) = 2000ms; echo time (TE) = 30ms; slices = 36; thickness = 4 mm; gap = 0 mm; field of view (FOV) = 240 mm × 240 mm; acquisition matrix = 64 × 64; and flip angle (FA) = 90°. The fMRI sequence took 8 min and 8 s. Three-dimensional turbo fast-echo (3D-TFE) T_1_WI sequence with high resolution: TR = 8.1mm; TE = 3.7 ms; slices = 170; thickness = 1 mm; gap = 0 mm; FA = 8°; acquisition matrix = 256 × 256; FOV = 256 mm × 256 mm; Fluid-attenuated inversion recovery (FLAIR): TR = 7000 ms; TE = 120ms; slices = 18; slice thickness = 6 mm; gap = 1.3 mm; FA = 110°; voxel size = 0.65 × 0.95 × 6 mm^3^.

### Image processing

Data analyses were preprocessed using Data Processing Assistant for Resting-State fMRI programs[15], which is based on Statistical Parametric Mapping (SPM8, http://www.fil.ion.ucl.ac.uk/spm) and resting-state fMRI data analysis toolkit (REST, http://www.restfmri.net). The first 10 volumes were discarded and the remaining 230 consecutive volumes were used for data analysis. Slice-timing and realignment for head motion correction were performed. Subjects with a head motion greater than 2.0 mm translation or a 2.0° rotation in any direction were excluded. Data were spatially normalized to the Montreal Neurological Institute template (resampling voxel size = 3 × 3 × 3 mm^3^), smoothed with an isotropic Gaussian kernel [full width at half maximum (FWHM) = 4 mm], detrended and filtered (0.01–0.08 Hz). The bilateral amygdala were set as seed regions using the WFU_PickAtlas software (http://www.ansir.wfubmc.edu). Effective connectivity was analyzed using REST-GCA in the REST toolbox [16]. In this study, time series of bilateral amygdala were defined as seed time series x, and time series y denoted all voxels in the brain. The linear direct influence of x on y (F x→y) and the linear direct influence of y on x (F y→x) were calculated voxel by voxel across the brain. Afterwards, two Granger causality maps were generated based on the influence measures for each subject. The residual-based F was normalized (F’) and standardized to Z score for each voxel (Z_x→y_ and Z_y→x_, subtracting the global mean F’values, divided by standard deviation).

### Statistical analysis

For group level analyses on effective connectivity of amygdala, mean values of Zx→y and Zy→x maps were calculated for each group. All eight Granger causality maps were acquired, with four for each direction and four for each group (left amygdala with Zx→y and Zy→x and right amygdala with Zx→y and Zy→x for both the patients and healthy controls). These Granger causality maps were entered into a voxel-wise two-sample t-test to determine the difference between MwoA patients and HCs with age, sex, and education included as covariates. For multiple comparison correction, Gaussian Random Field (GRF) with *p* < 0.01 (Z > 2.58) for voxel level results and *p* < 0.05 for cluster level was used. The threshold of cluster size was set at 118 voxels. Positive clusters based on RESTplus were generated as binary mask, and connective strengths of the significant regions were extracted based on z-maps.

Comparison of demographic data between MwoA patients and HCs were analyzed by using between-group t-test for means and 2-test for proportions (*p* < 0.05 was considered to be significant). To investigate the relation between fMRI data and clinical cognitive characteristic of MwoA, regions showing significant difference between groups were extracted. Then the mean z-values of aberrant functional connectivity region mask were calculated within each subject. Pearson’s correlation analysis between mean z-values of aberrant functional connectivity and each clinical cognitive characteristic were performed by using SPSS 17.0 (version 17.0; SPSS, Chicago, IL, USA). *P* < 0.05 was considered as statistically significant, corrected for age, sex and years of education. Bonferroni correction was used for multiple comparisons in correlation analyses.

## Results

### Demographic and clinical variables

As shown in Table 1, MwoA patients and HCs showed no significant difference in gender(*P* = 0.482), age(*P* = 0.108), MoCA score(*P* = 0.069), HAMA score(*P* = 0.334), HAMD score(*P* = 0.532), or years of education(*P* = 0.233).

**Table 1:** Demographic characteristics of the patients with MwoA and the healthy controls

	MwoA patients(*n* = 45)	HCs (*n* = 40)	*P* value
Age(years)	38.62 ± 10.11	35.45 ± 7.53	0.108
Gender(male/female)	12:33	14:26	0.482
MoCA score	27 ± 1.03	27.73 ± 1.93	0.069
HAMA score	37.16 ± 8.68	35.5 ± 6.78	0.334
HAMD score	41.76 ± 8.93	40.60 ± 7.92	0.532
Education(years)	14.36 ± 2.72	15.03 ± 2.38	0.233
Duration(years)	13.8 ± 6.07	NA	NA
Frequency(d/m)	4.31 ± 4.34	NA	NA
HIT-6 score	58.80 ± 8.09	NA	NA
MIDAS score	12.29 ± 6.95	NA	NA

Data are presented as mean ± SD; *MwoA* Migraine without aura; *HCs* Healthy controls; *MoCA* Montreal Cognitive Assessment; *HAMA* Hamilton Anxiety Scale; *HAMD* Hamilton Depression Scale; *d/m* day per month; *HIT-6* Headache Impact Test-6; *MIDAS* Migraine Disability Assessment Questionnaire

### Granger causality analysis

**Fig. 1:**
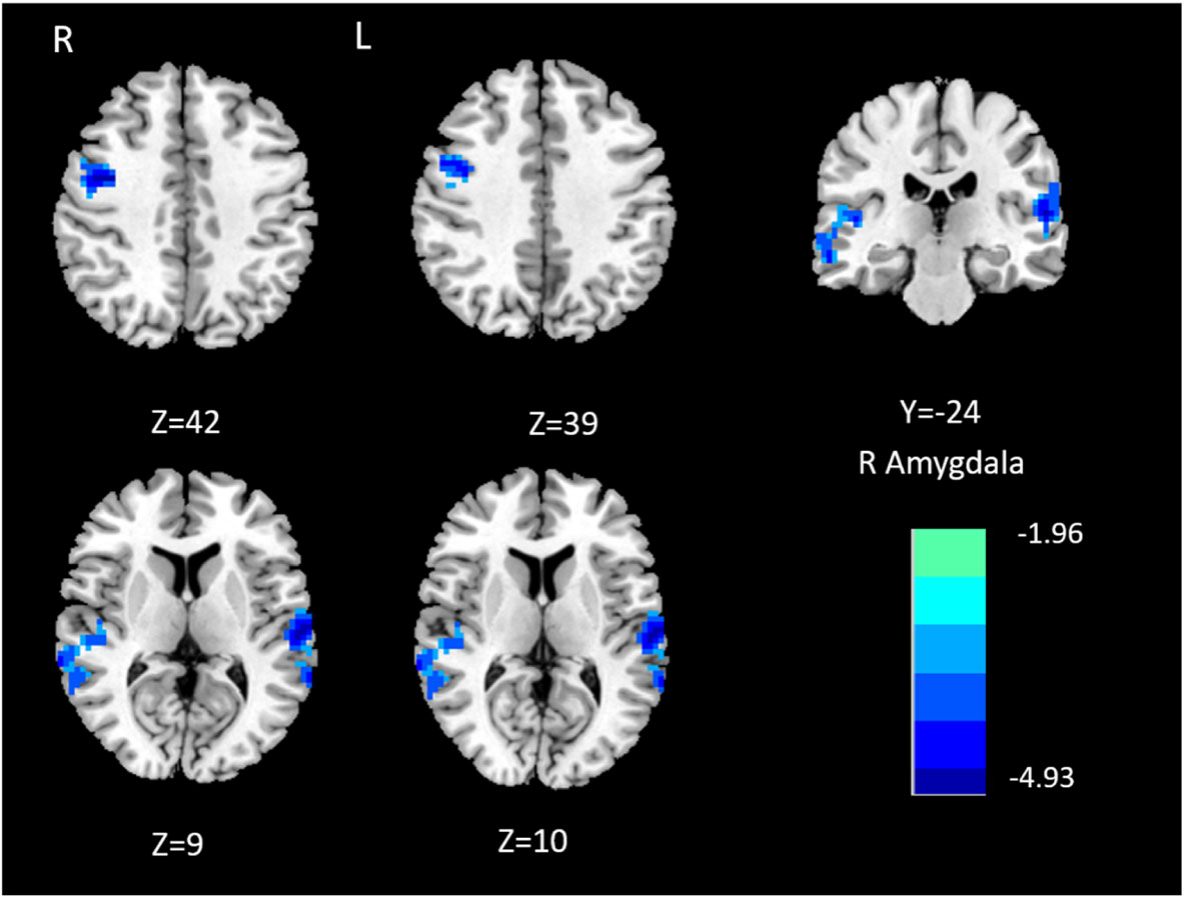
Causal outflow from R amygdala to the rest of the brain (X to Y)

**Fig. 2:**
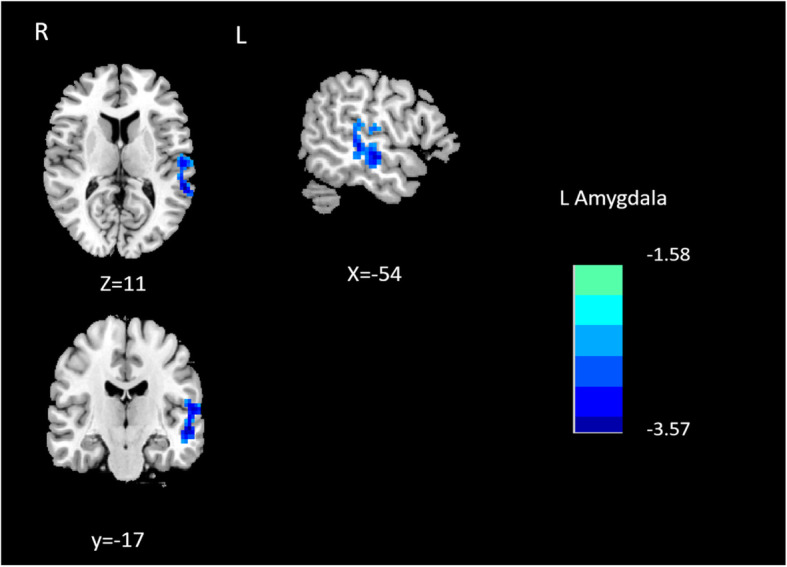
Causal outflow from L amygdala to the rest of the brain (X to Y)

**Fig. 3:**
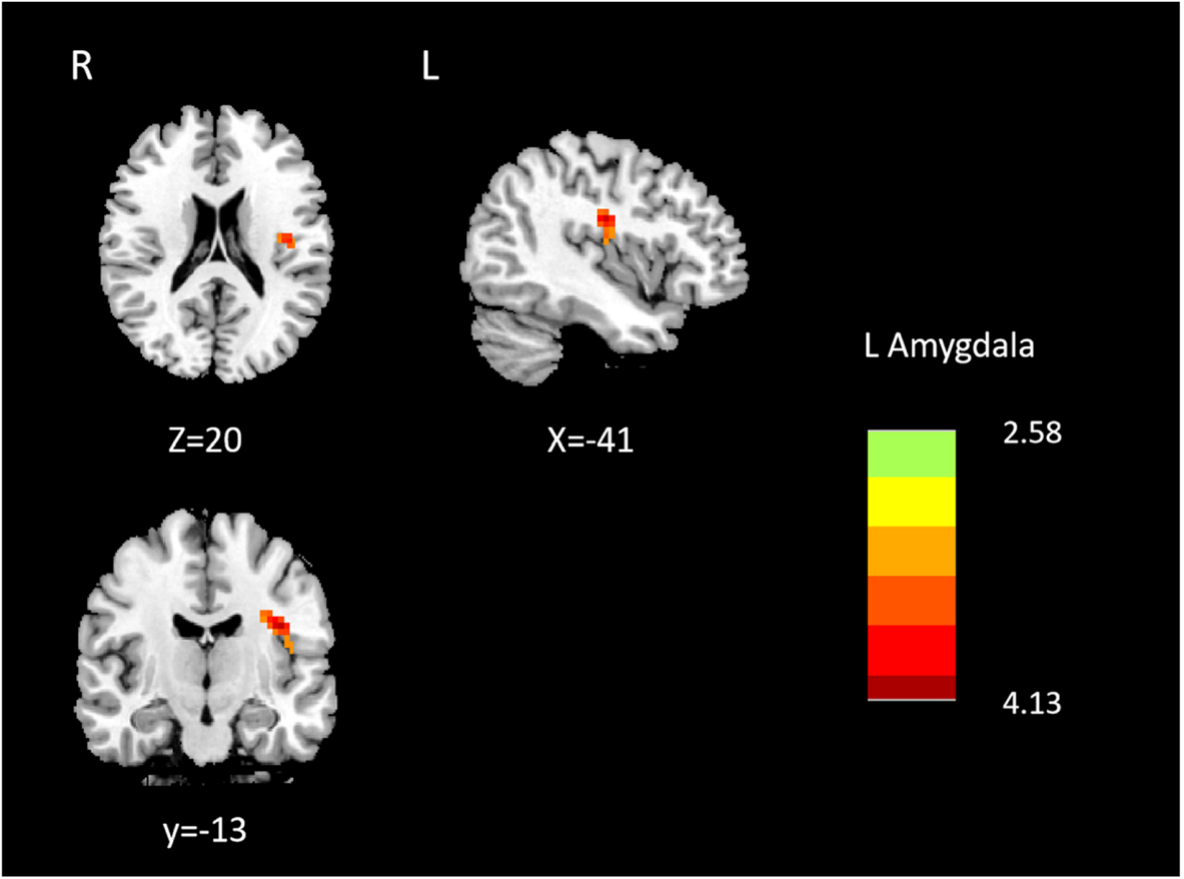
Causal inflow to L amygdala from the rest of the brain (Y to X)

MwoA patients showed significantly decreased effective connectivity from right amygdala to several brain regions that include right superior temporal gyrus, left superior temporal gyrus and right precentral gyrus compared with HCs (Table 2; Fig. 1). Furthermore, MwoA patients demonstrated significantly decreased effective connectivity from the left amygdala to the ipsilateral superior temporal gyrus (Table 3; Fig. 2). Also, MwoA patients showed enhanced effective connectivity from left inferior frontal gyrus to left amygdale (Table 3; Fig. 3).

**Table 2:** Two-sample t-test (voxel-level *P* < 0.01 and cluster-level *P* < 0.05 Gaussian random field corrected) of difference in causal influence to and from the right amygdala in patients with migraine without aura versus healthy controls

	Brain region	Peak MNI coordinates			Voxel size	Peak t score
		X	Y	Z		
Causal outflow from R amygdala to the rest of the brain (X to Y)						
	L_Sup_Temporal	-66	-48	12	214	-4.7341
	R_Sup_Temporal	54	-12	-6	366	-4.3447
	R_Precentral	42	-3	45	136	-4.9252
Causal inflow to R amygdala from the rest of the brain (Y to X)						
	-	-	-	-	-	-

*L* left; *R* right; *Sup* superior

**Table 3:** Two-sample t-test (voxel-level *P* < 0.01 and cluster-level *P* < 0.05 Gaussian random field corrected) of difference in causal influence to and from the left amygdala in patients with migraine without aura versus healthy controls

	Brain region	Peak MNI coordinates			Voxel size	Peak t score
		X	Y	Z		
Causal outflow from L amygdala to the rest of the brain (X to Y)						
	L_Sup_Temporal	-60	-42	12	203	-3.5682
Causal inflow to L amygdala from the rest of the brain (Y to X)						
	L_Inf_Frontal	-39	-15	-24	118	6.0593

*L* left; *Sup* superior; *Inf* inferior

### Correlation analysis

**Fig. 4:**
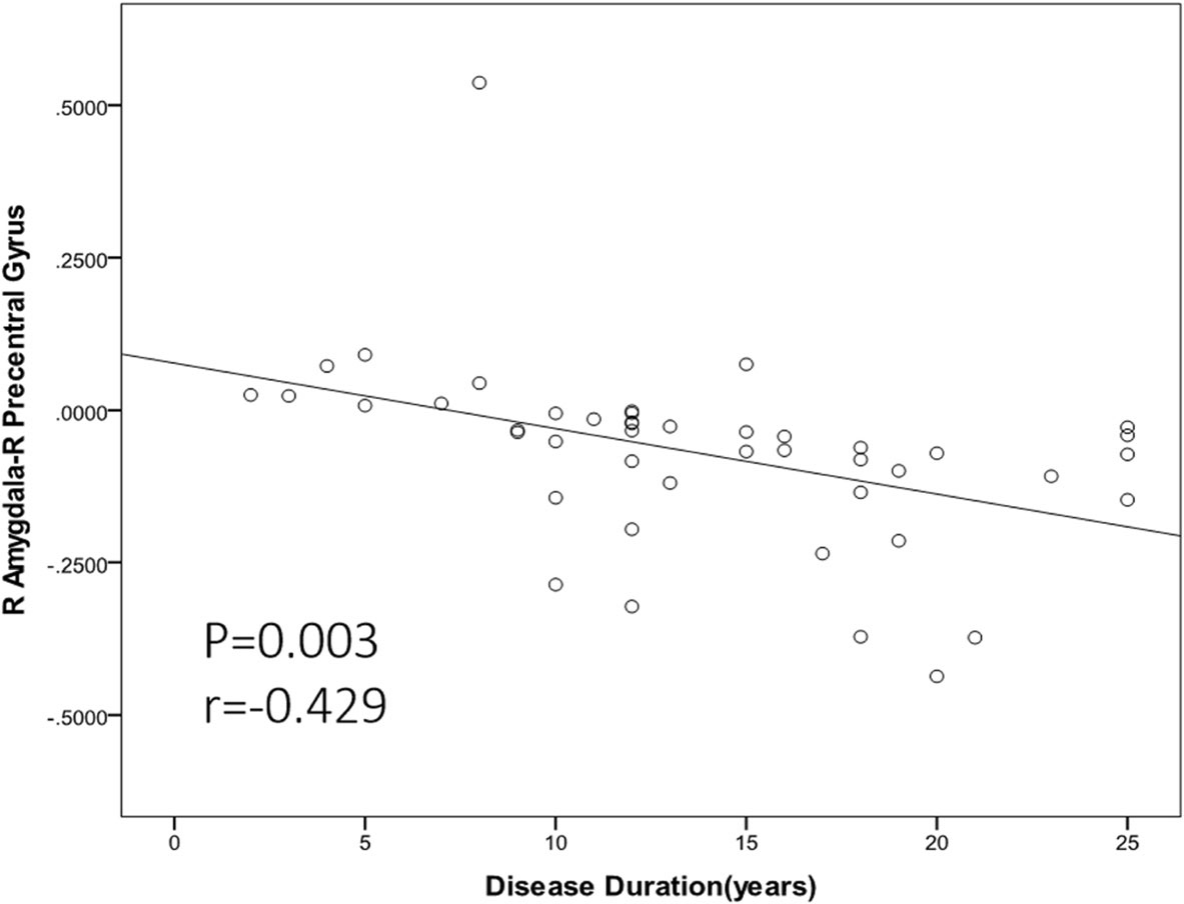
Correlations between effective connectivity from right amygdala to right precentral gyrus and disease duration

Effective connectivity outflow from right amygdala to right precentral gyrus was negatively correlated to disease duration (Fig. 4, *P* = 0.003, *r* = -0.429). No other significant linear correlation was observed.

## Discussion

The relation between limbic system and pain always receives much concern[17–19]. As an essential element of the limbic system, amygdala is worth of further study in migraineurs. To our knowledge, this is the first study to use GCA to identify changes in effective connectivity (directional connectivity) in amygdala which is strongly implicated in migraine. Our main finding showed that MwoA patients had significant changes in effective connectivity in a set of brain regions including diverse functional areas emanating from and projecting to the amygdale.

Our study indicated that there is a significantly decreased effective connectivity from the right amygdala to the bilateral superior temporal gyrus. During interictal period, MwoA patients might still suffer hypersensitivity to auditory, olfactory, or visual stimuli. The superior temporal gyrus is generally considered to be important as auditory perception and emotional regulatory part in human brain[20–23], which is essential for individual stressful experiences, cognitive processes and adaptive behavior. The amygdala is capable of modulating auditory cortex activity and plasticity that allows a particular individual to respond to the environment in a predictive manner. Some previous studies have shown that migraineurs, in the interictal phase and in migraine attack, have abnormalities in the function of neural substrates, responsible for different stages of temporal processing, especially in temporal resolution and temporal ordering[24–27]. Our results of decreased connectivity in each hemisphere from amygdala to superior temporal gyrus in MwoA patients may represent temporal processing impairment, which could be a reasonable explanation for photophobia and phonophobia linked to lower threshold for tolerating unpleasant signals. The superior temporal gyrus plays other neurocognitive functions, such as speaking and language recognition. In our study, MwoA patients showed decreased scores of visuospatial and executive function, name recognition and language compared with HCs (Table 4). Decreased superior temporal gyrus connectivity represented naming and language defects to some extent.

**Table 4:** Detailed category statistical analysis according to MoCA

	MwoA patients(n = 45)	Healthy controls(n = 40)	*P* value
Visuospatial and executive function	2.37 ± 0.451	4.67 ± 0.867	0.021
Name recognition	1.21 ± 0.532	2.95 ± 0.284	0.014
Attention	5.77 ± 0.721	5.85 ± 0.495	0.432
Language	1.81 ± 0.412	2.92 ± 0.195	0.032
Abstract	1.79 ± 0.431	1.93 ± 0.503	0.851
Delayed memory	3.45 ± 1.369	3.73 ± 1.325	0.654
Orientation	5.81 ± 0.507	5.97 ± 0.313	0.221

Migraine patients also showed decreased functional connectivity from the left amygdala to the left superior temporal gyrus. This laterality pattern may be associated with the different functions of bilateral amygdala, and the left amygdala was known to contribute to the brain’s reward system[28]. Therefore, the laterality mechanism should be further investigated.

Another finding of the present study was the decreased connectivity from the right amygdala to the right precentral gyrus. The effective connectivity outflow from right amygdala to the right precentral gyrus was negatively correlated with migraine duration. The precentral gyrus, which is part of the supplementary motor area(SMA), predominantly participants in the internally generated planning of movement, the planning of sequences of movement, also involved in pain anticipation. The reduced connectivity from the right amygdala to right precentral gyrus may be associated with disrupted projections and modulation of pain perception[17]. Since physical movement aggaravated the pain of migraine, less activation of precentral gyrus in MwoA at interictal period might indicate protective adaptive plasticity alteration after repetitive movement related pain experience. Our findings of reduced connectivity within SMA are in line with those of other studies showing decreased regional homogeneity and cortical thinning in migraineurs[29–31]. We speculate that nociceptive input modifies the functional patterns of the SMA and that these changes may account for the functional impairment in migrainuers.

Inferior frontal gyrus not only plays a role in cognitive modulation of pain, but also is involved in memory retrieval and emotional pain regulation [32]. Previous studies also showed aberrant cerebral perfusion changes[33] in the inferior frontal gyrus in migraineurs. Prior resting-state fMRI study also provided further support linking migraine physiopathology with the inferior frontal gyrus[34]. The enhanced effective connectivity from left inferior frontal gyrus to left amygdala might indicate maladaptive brain response due to repeated stress or unpleasant exposure in migraineurs. As such, migraine can be regarded as the consequence of multisensory interactions between pain modulation and limbic network.

Our study has several potential limitations. First, the moderate small sample size may have reduced our ability to detect causal relationships between abnormal effective connectivity and migraine characteristics. Furthermore, we examined patients only in the interictal phase, and therefore effective connectivity in the ictal phase must also be explored. Finally, the seed used in this study covered the whole amygdala; however the amygdala was subdivided into the basolateral nucleus group and the medial cortex group. Different parts may have their own distinct impact; therefore more accurately defined anatomical parcellation may influence the final results. These issues will be addressed in subsequent studies.

## Conclusions

Despite these limitations, our results identified disrupted effective connectivity networks in the amygdala of migraine patients. These findings may be associated with multisensory integration abnormalities and deficits in pain modulation that play a key role in the clinical characteristics in migraineurs, which could help enhance our understanding of the neuropathological mechanism underlying migraine.

## Data Availability

All data and materials generated in this study are available upon request.
